# Evaluation of the modified Wells score in predicting venous thromboembolic disease in patients with tuberculosis or HIV in a South African setting

**DOI:** 10.4102/sajhivmed.v23i1.1349

**Published:** 2022-03-23

**Authors:** Tweedy Keokgale, Sarah A. van Blydenstein, Ishmail S. Kalla

**Affiliations:** 1 Department of Internal Medicine, Chris Hani Baragwanath Academic Hospital, Johannesburg, South Africa; 2 Faculty of Health Sciences, University of the Witwatersrand, Johannesburg, South Africa; 3 Division of Pulmonology, Department of Internal Medicine, Chris Hani Baragwanath Academic Hospital, Johannesburg, South Africa; 4 Division of Critical care, Charlotte Maxeke Johannesburg Academic Hospital, Johannesburg, South Africa

**Keywords:** modified Wells score, HIV, tuberculosis, pulmonary embolism, deep vein thrombosis

## Abstract

**Background:**

There is paucity of data on the modified Wells score (MWS) utility on patients with venous thromboembolism (VTE) in a South African setting where there is a high burden of HIV and tuberculosis (TB). This study analyses the performance of this score in HIV/TB-infected patients compared with non-infected patients.

**Objectives:**

To assess the performance of the MWS as an additional risk factor for VTE in hospitalised patients with a high burden of HIV/TB infections.

**Method:**

This study was a retrospective cross-sectional cohort analysis of the utility of the MWS in 156 HIV/TB-infected and non-infected adult patients diagnosed with VTE on compression ultrasonography (CUS) or computed tomography pulmonary angiography (CTPA) in a medical inpatient setting over six months. Patients with HIV and/or TB were assessed as having an additional risk factor (1 point for each), and this was compared with the MWS. A McNeymar’s paired sample chi-squared test was used to compare the sensitivity of this score against the MWS.

**Results:**

Of the 156 patients with VTE who were enrolled, HIV was the commonest risk factor (42.31%) with TB accounting for 10.90% of cases. When the MWS adjusted for HIV/TB was used, the sensitivity increased from 25% to 100% for the HIV–/TB+ category, it increased from 77.36% to 98.11% in the HIV+/TB– category and it increased from 84.62% to 92.95% in the HIV+/TB+ category. These differences were statistically significant at *P* < 0.05 in all categories.

**Conclusion:**

The MWS performs better when the infectivity of HIV/TB is included as an additional risk factor in the score.

## Introduction

The modified Wells score (MWS) has been validated in determining the probability for venous thromboembolism (VTE) in multiple studies in high-income countries with a low prevalence of HIV and tuberculosis (TB).^1,2,3^ These infectious diseases do not appear as independent risk factors in the MWS. This prediction score is based on non-invasive clinical parameters that are derived from the history and examination of patients with VTE; each clinical parameter is allocated points that are added together to calculate the MWS (see Appendix 1 and Appendix 2). South Africa has a high prevalence of both HIV and TB,^4^ and the clinical utility of the MWS within such an environment has not been critically evaluated with HIV/TB as an additional risk factor.

Deep vein thrombosis (DVT) and/or pulmonary embolism (PE) are collectively known as VTE.^5^ Venous thromboembolism is the abnormal formation of clots in the venous system from an acquired or hereditary cause.^6^ The diagnostic approach to a patient with VTE includes the use of the MWS and D-dimer testing.^1^ In 1997, the Wells score was developed as a nine-component clinical prediction rule for DVT, with two points being deducted if an alternative diagnosis to DVT is at least as likely. This gives a possible score range of –2 to 8. There are three risk categories, namely high (≥ 3 points), intermediate (1–2 points) and low (< 1).^7,8^ In 2003, a further component, that is, previously documented DVT, was added to the original Wells score, while the duration of surgery was increased from 4 to 12 weeks.^1^ This gives a possible score of –2 to 9. This version of the MWS reduced the risk categories from three levels (e.g. low, intermediate and high) to two levels: likely (2 points or more) and unlikely (less than 2 points).^1^ In 1998, a seven-component clinical prediction rule was developed for PE: points are based on criteria in the history and on examination giving a possible score range of 0.0–12.5 with a score of > 6 predicting a high risk for PE, a score of 2–6 predicting an intermediate risk and a score of < 2 predicting a low risk.^8,9^ In 2000, the Wells score for PE was further revised reducing the number of risk categories to just two as for DVT: likely = > 4 points and unlikely = ≤ 4 points.^8,10^

Compression ultrasound (CUS) for DVT and computed tomography pulmonary angiography (CTPA) for PE can safely be withheld in patients who are unlikely to have VTE according to the MWS and a normal D-dimer.^2,3^ Patients who have a high MWS need a confirmatory CTPA or CUS for PE and DVT, respectively. The MWS is the most widely used score for VTE^4,11^ but lacks validation in a South African context with a high prevalence of HIV and TB infections.^12,13,14^ However, neither is part of the pretest probability score. Furthermore, within the South African context, HIV and TB infections are commonly regarded as two of the most important contributors to the rising numbers of VTEs.^15^ The current mortality associated with VTE in South Africa is approximately 20 000 deaths per annum.^15^ Three-quarters of these deaths occur in medically ill patients^16^ but the true prevalence of VTE in South Africa is unknown.^17^

### Aetiology of venous thromboembolism

Pulmonary embolism (PE) refers to a blockage of a major vessel in the lungs because of a thrombus.^18^ Deep vein thrombosis is often the precursor of a PE and is found in 70% of patients with PE.^19^ Venous thromboembolism is associated with significant morbidity and mortality from cardiac and pulmonary complications, hence the importance of an early diagnosis.^13^ Virchow’s triad describes three factors that contribute to the development of VTE, namely hypercoagulability, stasis and endothelial injury.^20^ The two most important categories of VTE risk factors are patient and procedure.

Patient-related risk factors include the following: age > 60 years, history of VTE, immobility, underlying malignancy, pregnancy, oestrogen therapy in the form of contraception and hormonal therapy, smoking, congestive heart failure, obesity, hereditary thrombophilic states, inflammatory bowel disease, nephrotic syndrome especially with chronic renal failure (chronic kidney disease [CKD]), HIV/TB and autoimmune diseases including anti-phospholipid syndrome.^16,21,22^

Procedure-related risk factors include the following: duration of the procedure, degree of tissue damage especially orthopaedic, degree of immobility following surgery and the nature of the surgical procedure.^16^

### Venous thromboembolism and HIV

There is a high burden of HIV in South Africa, with an estimated prevalence of 7.52 million people in 2018, of whom about 62% are on treatment, significantly below the target set by the World Health Organization (WHO).^23,24^ Although increasing age is a risk factor for VTE, the median age for VTE is about 40 years in a South African setting, potentially because of the high prevalence of HIV in this age group.^22,25^ Moodley et al. reported a median age of 40 years in HIV-infected VTE patients. HIV contributes to the development of a hypercoagulable state that predisposes to a twofold to tenfold increase in VTE patients versus the uninfected patients.^13^ In people living with HIV (PLHIV), there is disruption of the normal balance of coagulation factors with an increase in pro-thrombotic proteins such as von Willebrand factor and a reduction in naturally occurring anticoagulants such as protein S and protein C.^26^ While most abnormal coagulation factors improve after starting antiretroviral treatment (ART), the coagulopathy fails to normalise completely.^27^ People living with HIV also have higher levels of the lupus anticoagulant, homocysteine, anti-cardiolipin and anti-phospholipid antibodies than the general population; these factors also contribute to a pro-thrombotic state.^26^ In addition, HIV may directly damage vascular endothelium rendering the vessel wall pro-thrombotic.^13^

Similarly, opportunistic infections (OIs) including cytomegalovirus (CMV), pneumocystis pneumonia (PJP) and Mycobacterium avium complex (MAC) have been associated with VTE.^13^ Furthermore, antiretroviral drugs such as protease inhibitors (PIs) promote thrombosis via an effect on the metabolism of thrombotic proteins in the liver.^13^ A systemic literature review of 13 studies between 1991 and 2007 reported an annual incidence of VTE between 0.19% and 7.73% in PLHIV per year.^13^ Low CD4 cell counts and malignancy are reported as other important risk factors for VTE.^11^

### Venous thromboembolism and tuberculosis

According to the 2018 WHO’s Global Report, approximately 322 000 South Africans contracted TB in 2017.^28^ A 2010–2011 study from Dr George Mukhari Academic Hospital (Pretoria) reported a 47.00% prevalence of TB in patients with VTE.^29^ In an audit of VTE in a Johannesburg hospital, the prevalence of HIV was 50% and that of TB was 30.00%.^22^ Awolesi et al. reported a similarly high prevalence of 51.85% and 35.80% for HIV and TB, respectively, in their cohort of patients in KwaZulu-Natal.^25^ Indeed, these researchers point out that TB is the commonest OI of PLHIV, associated with an increased risk for VTE.^25^ Tuberculosis induces a pro-thrombotic state via the production of cytokines, such as tumour necrosis factor alpha (TNF-α) and interleukin 6 (IL-6), that render vascular endothelium thrombogenic. HIV interferes with the production of hepatic coagulation factors in the liver increasing factor VIII, fibrinogen and plasminogen activator inhibitor 1 (PAI1), and reducing antithrombin and protein C levels.^12,30,31^ The causal relationship between TB and VTE is also demonstrated in the improved pro-thrombotic state a month after initiating TB therapy and the introduction of rifampicin that induces the hepatic coagulation protein synthesis and increases the risk of thrombosis.^31,32^

### Diagnosis of venous thromboembolism

Appendix 3 illustrates the diagnostic algorithm for suspected VTE.^5^ Evidence-based literature supports the practice of determining clinical probability.^33^ Patients with a high MWS (≥ 2 for DVT and ≥ 5 for PE) are subjected to gold standard testing with CUS and CTPA for DVT and PE, respectively (see Appendix 3); if the score is low, blood is drawn for D-dimers.^8^

Multiple studies have shown that VTE can safely be ruled out by low probability MWS and a normal D-dimer test.^1,2,3^ A normal D-dimer level is 0.0 mg/L – 0.25 mg/L.^34^ However, if a patient is older than 50 years, an adjusted D-dimer must be used (age multiplied by 10 ng/mL) as D-dimers levels increase with age.^35^ If the D-dimers levels are elevated, the patient is subjected to a gold standard confirmatory test (Appendix 3).

### Accuracy of the Wells score

Wells et al. previously published studies reporting the incidence of PE to be 5.00% – 8.00% and 39.00% – 41.00% in the unlikely and likely groups for MWS, respectively.^10^ For DVT, Wells et al. when evaluating the 3-level Wells score 3.00%, 17.00% and 75.00% of the patients with low, moderate and high pretest probability, respectively, had DVT.^7^ Subsequently, multiple studies have confirmed the accuracy, efficiency and sensitivity of the Wells score with published sensitivity of 92.00% by Rabab et al. and 95.00% by Amit Bahia for PE and 76.10% sensitivity for DVT.^36,37,38^ Modi et al. reported a specificity, sensitivity, positive predictive value and negative predictive value of 90.00%, 67.00%, 31.00% and 98.00%, respectively, for DVT using the 3-level score. In the 2-level score, sensitivity, specificity, positive predictive value and negative predictive value were 100.00%, 36.00%, 9.00% and 100.00%, respectively.^39^ Lucassen et al. reported a meta-analysis with a sensitivity of 84.00% and specificity of 58.00% with the 3-level score,^40^ while with the 2-level score the sensitivity and specificity were 60.00% and 80.00%, respectively, for PE while Bahia et al. reported a specificity of only 27.00%.^36^ A prospective validation study by Wolf et al. found kappa values for Wells criteria to be 0.54 and 0.72 for the 3-level and 2-level scorings, respectively.^41^ H’Ng et al. reported the 2-level Wells score to have a specificity of 57.74% and sensitivity of 78.57% for DVT^42^ approximating that to the MWS by Subramaniam et al. which showed 75.00% sensitivity and 55.00% specificity.^43^ Agreement between a positive Wells score and radiological results for VTE was significant in Owaidah et al.’s study showing sensitivity of 88.00%, specificity of 55.00%, positive predictive value of 26.00% and negative predictive value of 96.00%.^34^ Given the above literature, the true sensitivity and specificity of the Wells score are unclear as the studies were all done under different settings and permutations (outpatient, trauma, emergency department, inpatient), and some studies assessed the ‘old’ 3-level score while other studies assessed the 2-level MWS.

### Wells criteria use in HIV/TB

There is no data that validate the MWS in countries with a high burden of HIV and TB. The MWS has been validated with traditional risk factors in high-income countries.^1,44^ Given the high burden of HIV/TB in South Africa, there is a concern that the MWS might not perform as it does in assessing the probability of VTE in a non-HIV/TB population as compared with the HIV/TB-infected patients.

Considering the poor sensitivity of D-dimers as a rule-in test in the face of HIV/TB co-infection, should we consider HIV/TB an additional risk factor for the MWS? This is being hypothesised in South Africa as many studies have shown that the commonest cause for VTE is HIV/TB.^22,25,29,45^ A recommendation by Mampuya et al. suggests that doctors working in a primary setting should be trained in the prompt diagnosis and early management of VTE.^45^ Both Mampuya et al. and Awolesi et al. concluded that a scoring system that includes HIV/TB should be considered in the South African setting so that gold standard tests are ordered promptly without awaiting D-dimers if the MWS probability is high.^25,45^ Their studies showed that the MWS diagnostic accuracy is improved when using a score that includes HIV/TB as independent risk factors.

## Methodology

### Research question

To assess the accuracy of the MWS in HIV- and TB-infected patients in a South African cohort of inpatients as compared with the findings of the current published accuracy of this prediction rule.

### Objectives

To compare the gold standard imaging confirmed on CUS and CTPA positive results for VTE with the MWS.
To compare the performance of this prediction rule in a cohort of patients with the following permutations:
  HIV negative and TB negative (control) versus:
  ■ HIV-positive and TB positive
  ■ HIV-positive and TB negative
  ■ HIV-negative and TB positive
Compare the performance of the MWS with a score that includes HIV/TB as additional risk factors. A score of one each will be allocated for HIV and TB.

### Research design

This study was a retrospective cross-sectional cohort analysis of 156 adult patients who were diagnosed with VTE at Chris Hani Baragwanath Academic Hospital (CHBAH) and Sebokeng Regional Hospital (SBH) to determine the predictability of the MWS. Adult patients with confirmed VTE on CUS or CTPA were included. Patients were excluded if VTE was diagnosed by ventilation-perfusion scan (V/Q), if they had unconfirmed HIV status, if they were pregnant and if they had undergone surgery.

### Data collection

Data collection was performed by the principal investigator (PI) via face-to-face interaction. Patients were recruited over a period of 6 months. Patient records were reviewed to complete the datasheet (Appendix 4), Appendix 1 and Appendix 2 (MWS). History and examination were performed by the PI to ascertain the clinical features that appear in the MWS, and TB had to be definitively diagnosed as per the following:

- GeneXpert (nucleic acid amplification test for *Mycobacterium tuberculosis* and rifampicin sensitivity) on sputum, pleural fluid, ascitic fluid, cerebrospinal fluid, bronchial washings or other specimens
- Acid fast *Bacilli* (AFB) on specimen
- Compatible histology, that is, granulomas with caseous necrosis
- Urinary antigen detection of lipoarabinomannan (LAM)
- Culture of TB with or without drug sensitivity
- Patients that are screened negative as per the Gauteng Hospital protocol were treated as negative for TB as further testing was not required.

### Data analysis

The information obtained from the datasheet was entered into an Microsoft Excel^®^ spreadsheet. Data were then exported to Stata version 15, a software program, for further analysis. The demographic and clinical characteristics of all patients were recorded. Categorical variables were described using frequencies and percentages. A bar graph was used to explore age distribution in patients with VTE. Patients with VTE were categorised into four groups: (1) HIV-negative and TB negative, (2) HIV negative and TB positive, (3) HIV positive and TB negative, and (4) HIV-positive and TB positive. Fisher’s exact test was used to compare differences in sensitivity of the MWS to correctly assign a positive VTE status across the four patient categories.

The overall sensitivity of the MWS, the adjusted MWS (HIV and/or TB) and D-dimers to correctly assign a positive VTE status was estimated using proportions with logit-transformed 95% confidence intervals. The McNemar’s paired sample chi-squared test was used to compare the sensitivity of each score against the MWS.

Patients were further categorised into cases (HIV positive and/or TB positive) and controls (HIV negative and TB negative). Differences in age, gender, MWS and D-dimers in cases and controls were explored. Student’s *t*-test for the comparison of means was used to compare normally distributed continuous variables (age and MWS), Wilcoxon rank-sum test for the equality of medians was used to compare D-dimers that were not normally distributed and Pearson’s chi-squared test was used to compare proportions by gender.

The mean MWS and the median D-dimers for each patient category are presented. Analysis of variance (ANOVA) was used to compare the mean MWS across patient categories. The Kruskal–Wallis equality-of-populations rank test was used to compare the median D-dimers by patient category.

### Ethical considerations

Ethics approval was obtained from the University of the Witwatersrand’s Research Ethics Committee (HREC Medical) (reference number: M190680). Permission for the use of patient records and patient interview was obtained from the Clinical Head of the Department of Internal Medicine and the Chief Executive Officer/Superintendent/Clinical Manager of the two hospitals (CHBAH and SBH). The research proposal was submitted for approval by the National Health Research Ethics Council (NHREC) (reference number: GP201910001).

## Results and discussion

### Incidence and demographics

A total of 156 patients were enrolled in this study (Table 1), and 72.44% of the patients were female (Table 1), in keeping with previous VTE studies demonstrating a female predominance.^25,45,46^ Stats SA reports that women are more likely to attend healthcare facilities earlier than men, which explains the female predominance.^23^ Other factors that could account for this are the use of oral contraception, hormone replacement therapy and pregnancy, which are proven risk factors for VTE.^21,22^

**TABLE 1:** Summary of the characteristics, D-dimer scores and modified Wells scores of patients included in this study with venous thromboembolism (*n* = 156).

Features	*n*	(%)	Mean	s.d.	Median	IQR
**Demographic features**						
**Age at presentation (years)**	-	-	51.54	17.91	-	-
< 20	3	1.92	-	-	-	-
20–29	13	8.33	-	-	-	-
30–39	33	21.15	-	-	-	-
40–49	26	16.67	-	-	-	-
50–59	25	16.03	-	-	-	-
60–69	28	17.95	-	-	-	-
70–79	17	10.90	-	-	-	-
≥ 80	11	7.05	-	-	-	-
**Gender**						
Male	43	27.56	-	-	-	-
Female	113	72.44	-	-	-	-
**Clinical features**						
**HIV**						
Negative	90	57.69	-	-	-	-
Positive	66	42.31	-	-	-	-
TB						
Negative	139	89.11	-	-	-	-
Positive	17	10.90	-	-	-	-
**Type of VTE**						
DVT	121	77.56	-	-	-	-
PE	31	19.87	-	-	-	-
DVT and PE	4	2.56	-	-	-	-
**Risk factors for VTE**						
HIV/TB	52	33.33	-	-	-	-
Metabolic syndrome	36	23.08	-	-	-	-
Malignancy	17	10.90	-	-	-	-
Unknown/none	14	8.97	-	-	-	-
Bedridden/immobility	9	5.77	-	-	-	-
Autoimmune disease	5	3.21	-	-	-	-
Hormonal contraceptive use	5	3.21	-	-	-	-
Sepsis/infection	5	3.21	-	-	-	-
Other	5	3.21	-	-	-	-
Smoking	4	2.56	-	-	-	-
Congestive heart failure	2	1.28	-	-	-	-
CKD	2	1.28	-	-	-	-
**Patient category**						
HIV-negative, TB negative	86	55.13	-	-	-	-
HIV-negative, TB positive	4	2.56	-	-	-	-
HIV-positive, TB negative	53	33.97	-	-	-	-
HIV-positive, TB positive	13	8.33	-	-	-	-
**D-dimers score**	-	-	-	-	2.89	1.18–5.80
**Modified Wells score**	-	-	-	-	4	3–5

TB, tuberculosis; VTE, venous thromboembolism; DVT; deep vein thrombosis; PE, pulmonary embolism; CKD, chronic kidney disease; IQR, interquartile range, s.d., standard deviation, n, number, CHBAH, Chris Hani Baragwanath Academic Hospital; SBH, Sebokeng Regional Hospital.

CHBAH, *n* = 137; SBH, *n* = 19.

We report a prevalence of 42.31% HIV-positive patients with VTE in our study (Table 1), with the current general HIV prevalence in South Africa reported at 13.10% in 2018.^23,47^ This increased prevalence of VTE supports the hypothesis that HIV is a major risk factor for VTE in the South African context.^29^ An audit by Louw et al. reported an HIV prevalence of 84.00% in patients with VTE.^12^

Of the 66 that tested HIV positive, 21 had other risk factors (excluding TB) (31.8%) (5 metabolic syndrome, 7 malignancy, 2 sepsis, 2 previous central lines, 2 combined oral contraception, 1 long-distance travel, 1 smoker and 1 end-stage renal disease), 13 had concomitant TB and 4 had TB alone without HIV. The only control was between non-HIV/TB infectivity and HIV/TB infectivity; it was difficult to find patients who had HIV/TB only, and 31.8% of them had other risk factors, so indeed we don’t know in those patients if the risk factor for VTE was HIV/TB or other.

The prevalence of VTE in patients that tested positive for TB in this study was 10.90% (Table 1). This prevalence is also much higher than the national prevalence of active TB reported to be 0.04% (322 000) in South Africa in 2017. A study at Dr George Mukhari Academic Hospital reported a 47.00% prevalence of TB in patients with VTE.^29^

In a 1-year audit of patients with VTE at a Johannesburg hospital, the prevalence of HIV was 50.00% and that of TB was 30.00%,^22^ with a similar finding by Alowesi et al. in a Kwazulu-Natal study that reported a prevalence of 51.85% and 35.80% for HIV and TB, respectively. These findings are discordant with this study where we report a prevalence of 42.31% for HIV and only 10.90% for TB (Table 1). The reasons for this discrepancy are not clearly evident from the data analysed; however, some of the reasons might be the time period of data collection, the setting of the study and the exclusion of patients where a ventilation perfusion scan (V/Q scan) was used to diagnose VTE. Another explanation could be the introduction of the antiretroviral programme that has seen some improvement over the years with more patients getting treated earlier than before. However, Mampuya et al. demonstrated a TB prevalence of 12.40%,^45^ similar to the findings in this study. For patients diagnosed with TB, the low prevalence of VTE in this study as compared with some of the other South African studies could be because of the strict inclusion criteria in which only patients with a confirmed laboratory diagnosis of TB were included in the analysis.

### Distribution of age in patients with venous thromboembolism

The distribution of age in our study has two peaks as shown in Figure 1 (age 30–39 years and 60–69 years). Advancing age is a risk factor for thrombosis in the developed world, but the mean age of HIV-infected patients at the time of VTE is 40 years.^47^ In our study, we demonstrated almost the same trend, and patients with HIV/TB were significantly younger than controls (non-infected): mean age 43.46 years (s.d.: 13.10) versus 58.13 years (s.d.: 18.65); *p* < 0.0001 (Table 2). This is possibly because of the high prevalence of HIV/TB in the younger population (age: 15–49 years).^23^

**FIGURE 1:**
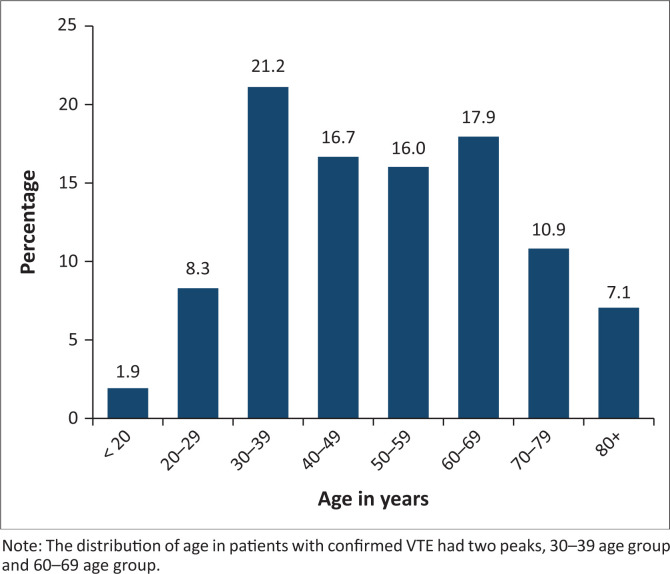
Distribution of age in patients with venous thromboembolism.

**TABLE 2:** Comparison of cases and controls (*n* = 156).

Variable	HIV-negative/TB negative (Controls)						HIV and/or TB positive						*p*
	Mean	s.d.	*n*	%	Median	IQR	Mean	s.d.	*n*	%	Median	IQR	
**Age**	58.13	18.65	-	-		-	43.46	13.10	-	-	-	-	< 0.0001*
**Gender**													
Male	-	-	22	51.16	-	-	-	-	21	48.84	-	-	-
Female	-	-	64	56.64	-	-	-	-	49	43.39	-	-	0.539
Modified Wells score	4.37	1.60	-	-	-	-	3.91	1.35	-	-	-	-	0.055
D-dimers	-	-	-	-	2.69	1.12–5.8	-	-	-	-	3.02	1.2–6.12	0.692

s.d., standard deviation; IQR, interquartile range; TB, tuberculosis.

* , Statistically significant.

### Performance of the modified Wells score with gold standard imaging

The MWS performed well, proving that it can be validated in a South African setting; of the 156 patients in the study, 130 patients were classified as high probability according to the MWS for VTE (83.33%); this is almost comparable with the published accuracy of 92.00% and 95.00% for PE and 76.10% for DVT.^36,37,38^ This finding supports the diagnostic algorithm of immediate imaging in the high probability patient as a confirmatory investigation.^7,9^ Our study also showed that the MWS has utility in the South African context; as the MWS performance was the same in HIV/TB-infected patients as compared with non-infected patients (Table 2 and Table 4), the mean MWS for controls and cases was 4.37 and 3.91, respectively (*p* = 0.055); hence, the difference was not statistically significant.

### Performance of the modified Wells score in the following categories

- I: HIV–/TB– (control)
- II: HIV+/TB+ or HIV–/TB+ or HIV+/TB–

Evidence-based literature supports the use of clinical probability scores to improve diagnostic algorithms. The MWS is one of the most validated and widely used scores.^10^ Multiple studies have been conducted to analyse the sensitivity, specificity and accuracy of the MWS. In a comparison of the Wells score and Doppler ultrasound in the diagnosis of DVT, the Wells score showed a sensitivity of 76.10%.^38^ For PE, the Wells score showed a sensitivity of between 92.00% and 95.00% when compared with CTPA.^36,37^ It needs to be noted, however, that the accuracy of the Wells score and its sensitivity are not clear because the studies that have been done are very heterogeneous in regard to the patient population selection and the setting in which the score was applied. This study was performed on medical inpatients with confirmed VTE on either CTPA or CUS. One of the studies that was similar to our study is by Owaidah et al. but they used the 3-level score and they reported a sensitivity of 88.00% and a specificity of 55.00% for DVT/PE combined,^34^ whereas our study showed a sensitivity of 83.33% for DVT/PE (Table 4), which is comparable even though we used different scores. In another study by Rabab et al., they demonstrated a 92% sensitivity in inpatients with PE using the MWS.^37^

There is a paucity of data in the literature that assess the accuracy of the MWS in a South African setting.

In our study, we report an average sensitivity of 83.33% for PE and/or DVT in all categories (Table 4). There were statistically significant differences in the sensitivity of the adjusted MWS by patient category (*p* < 0.05 in all categories) (Table 3). Sensitivity of the score for HIV–/TB– patients was 89.53%. For HIV+/TB– patients, the sensitivity was lower at 77.36%; for HIV+/TB+ patients, the sensitivity was 84.62%, which was lower than the control group and the published accuracy mentioned above. Lastly, for the group HIV–/TB+, the sensitivity was only 25.00% (Table 4).

**TABLE 3:** Comparison of the overall sensitivity of the modified Wells score versus the adjusted modified Wells score for HIV/tuberculosis as additional risk factors.

Scores	MWS (95% CI)	Comparison group		Difference		McNemar’s chi-squared test *p*-value
		MWS & HIV only	95% CI	Difference	95% CI	
MWS versus MWS + HIV only	76.66–86.43	91.02	85.36–94.63	7.69	2.86–12.51	0.0005*
MWS versus MWS + TB only	76.66–86.43	85.90	79.45–90.56	2.56	0.56–5.68	0.0455*
MWS versus MWS + HIV + TB	76.66–88.43	92.95	87.66–96.07	9.61	4.35–14.88	0.0001*

Note: MWS score - 83.33 (non adjusted for HIV/TB).

TB, tuberculosis; MWS, modified Wells score; CI, confidence interval; *p*-value, probability value.

* , Statistically significant.

**TABLE 4:** Sensitivity of the modified Wells score, before and after adjustment for HIV and/or tuberculosis.

Patient category	Modified Wells score		Modified Wells score adjusted for HIV+ only (+1)		Modified Wells score adjusted for TB+ only (+1)		Modified Wells score adjusted for HIV+ and TB+ (+1 +1)		Total
	*n*	%	*n*	%	*n*	%	*n*	%	
HIV-negative and TB negative	77	89.53	77	89.53	77	89.53	77	89.53	86
HIV-negative and TB positive	1	25.00	1	25.00	4	100.00	4	100.00	4
HIV-positive and TB negative	41	77.36	52	98.11	41	77.36	52	98.11	53
HIV-positive and TB positive	11	84.62	12	92.31	12	92.31	12	92.31	13

**Overall**	**130**	**83.33**	**142**	**91.03**	**134**	**85.90**	**145**	**92.95**	**156**

TB, tuberculosis; VTE, venous thromboembolism.

This clearly shows that the score underperforms in HIV/TB-infected patients. The reason for this could be that HIV and/or TB are not included in the score as independent risk factors, while there is actually a high burden of those two diseases in South Africa. Table 3 clearly shows the significance of including HIV/TB as additional risk factors. Furthermore, there are no data that confirm the validation of the MWS in South Africa, while it has been well validated in high-income countries with traditional risk factors.^25,45^ Nonetheless, it is hypothesised in multiple studies in South Africa that the commonest risk factor for VTE is HIV/TB,^44,48^ and indeed in our study, the commonest risk factor was HIV at 42.31% and TB at 10.90%, which is the third after HIV and metabolic syndrome, respectively (Table 1).

### Performance of the modified Wells score with a score that includes HIV/tuberculosis as additional risk factors

Mampuya et al. and Awolesi et al. concluded in their studies that a scoring system that includes HIV/TB should be considered in a South African setting so that gold standard tests are ordered promptly without awaiting D-dimers in the event that the probability is low according to the MWS.^25,45^ This is particularly important because D-dimers are a poor rule-in test when they are positive,^1^ and the studies by Mampuya et al. and Awolesi et al. showed that the MWS diagnostic accuracy is improved when using a score that includes HIV/TB as independent risk factors.

In our study, we report a similar outcome when the MWS is adjusted to include HIV and/or TB (+1 for each); the sensitivity increased from 25.00% to 100.00% for the HIV–/TB+ category, it increased from 77.36% to 98.11% in the HIV+/TB– category and it increased from 84.62% to 92.95% in the HIV+/TB+ category. The differences were all statistically significant at a *p*-value of < 0.05 for all categories (Table 3). The underdiagnosis using the unadjusted MWS has significant implications in that we are potentially missing VTE in HIV/TB-infected patients; does this mean the adjusted MWS can also be applied in HIV/TB low prevalent countries? Could the addition of HIV/TB as additional risk factors even in high-income countries be something to be considered? (Appendix 5).

An addition would improve the predictability in those countries; however, as the prevalence is low it is unclear if the change would be statistically significant because the commonest cause of VTE in those countries isn’t HIV/TB and its rather malignancy which is already included in the MWS.

The improved diagnostic accuracy of the MWS adjusted for HIV/TB means we can now rely less on D-dimers to diagnose VTE and rely more on the adjusted MWS as a pretest probability score. The current diagnostic algorithm used for VTE recommends that if the MWS is low and VTE is still suspected, one has to do a D-dimer; if the D-dimer is positive, only then can one request imaging. However, we report that in the event of a patient who has HIV/TB, an additional score of 1 (HIV or TB only) or 2, if both are positive, can allow the clinician to order imaging promptly without a D-dimer if the MWS is assessed as ‘likely’ for VTE. This can potentially save more lives as we can diagnose patients quicker which will lead to faster treatment. In a primary healthcare setting, this will allow patients to be transferred quickly to referral hospitals and potentially save money that might needlessly have been spent on D-dimers in the HIV/TB patient cohort where the D-dimer results are not always immediately available and have a poor sensitivity and a poor positive predictive value.

### Venous thromboembolism correlation with CD4 count

It has been shown that the lower the CD4 count, the greater the chance of having VTE.^13,49^ Explanation for this is that patients are more pro-thrombotic at lower CD4 counts.^13,49^ In our study, we report the same trend (Table 5); of the 66 patients that tested positive for HIV and had a confirmed VTE, 62 had CD4 count recorded and 51.61% had a CD4 count less than 200 cells/μL (median = 184; IQR = 74–540). Viral load suppression did not help with the prediction of VTE in our study because 65.08% of the patients with a measured viral load had levels that were suppressed (Table 5). This is in keeping with Bibas et al.’s study that showed that there is no correlation between viral suppression/non-suppression and thrombotic phenomenon.

**TABLE 5:** Summary of the CD4 and viral load results obtained for patients with venous thromboembolism in this study.

Variable	*n*	%	Median	IQR
**HIV prevalence in VTE patients (*N* = 156)**				
Negative	90	57.69	-	-
Positive	66	42.31	-	-
**CD4 count in HIV+ patients (*N*= 66)**				
CD4 count done	62	93.93	-	-
CD4 count not done	4	6.06	-	-
**CD4 count, cells/μL (*N* = 62)**				
< 200	32	51.61	-	-
200 to < 350	3	4.84	-	-
350 to < 500	7	11.29	-	-
> 500	20	32.26	-	-
Overall median CD4	-	-	184	74–540
Viral load, copies/mL (*N* = 63)				
Virally suppressed	41	65.08	-	-
Not suppressed	22	34.92	-	-
Overall median VL	-	-	61	0–23 100
Virally suppressed/CD4 > 200	26	41.27	-	-

VTE, venous thromboembolism; IQR, interquartile range; VL, viral load; CD4, cluster of differentiation.

### Strengths

An MWS that includes HIV and/or TB in the scoring system is inexpensive and fast, and it is a score that could potentially alter the prediction model when diagnosing VTE in an HIV/TB high-burden setting.

### Limitations

Colleague referrals and weekly screens were the methods of identifying patients. This could have impacted the number of patients included in the study with confirmed VTE for the period of data collection.

Most patients did not have MWS recorded in files, so the investigator retrospectively calculated the score in confirmed cases, which could have introduced bias to this study.

Tuberculosis cases enrolled for the study had to have a microbiological confirmation of TB.

Venous thromboembolism patients diagnosed with VQ scan were not enrolled in the study as this method of confirmation is not universally regarded as a gold standard for the diagnosis of PE, and this could have decreased the patient recruitment number as well.

### Recommendations

For easier data collection, we recommend that the PI make use of the Department of Radiology to identify all patients with a confirmed VTE diagnosis.

Before data collection, we suggest an algorithm for the diagnosis of VTE be made available to admitting doctors so that all patients can have their MWSs recorded to avoid bias by the investigator. We recommend the use of the MWS as it has been well validated and its usefulness confirmed in this study.

Based on the results:

- We recommend that a score that includes HIV/TB infections as additional independent risk factors be considered with further studies over a longer period of time to obtain an improved analysis.
- We recommend that studies be performed to assess if thrombo-prophylaxis should be considered in all HIV/TB-infected patients.
- We recommend that a consideration is made to include all TB cases in the study regardless of the method of diagnosis.

## Conclusion

The MWS has not been validated in a South African setting where there is a high burden of HIV/TB. This study has shown that the MWS is reliable in the South African context; however, its accuracy is improved when adjusted to include HIV and/or TB as additional independent risk factors.

## Figures and Tables

**FIGURE 1-A1:**
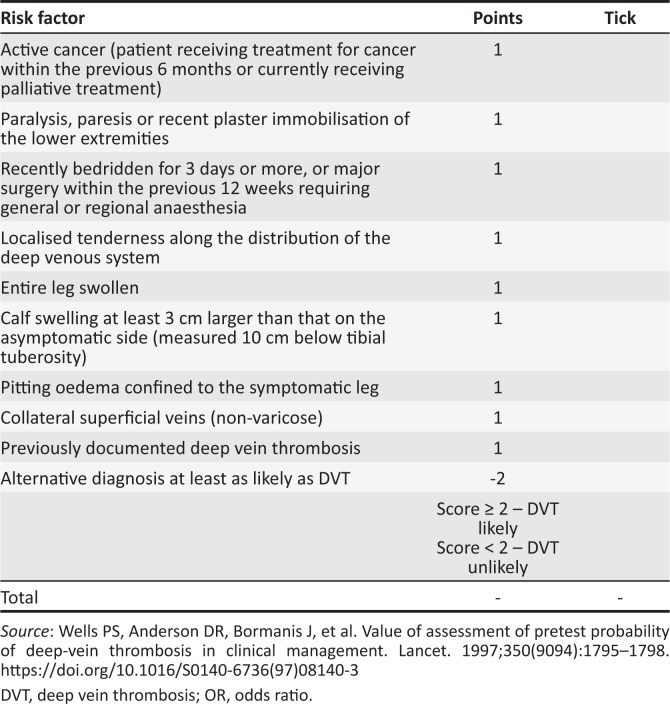
Modified Wells score for DVT.

**FIGURE 1-A2:**
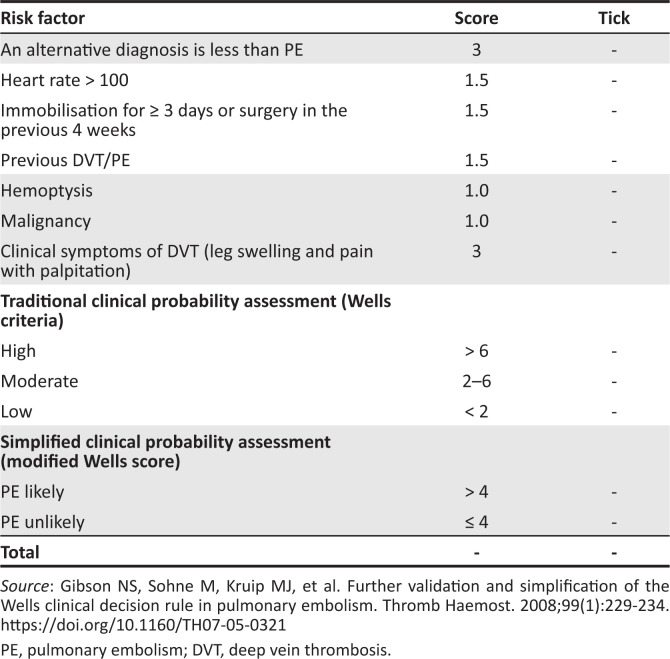
Modified Wells score for pulmonary embolism.

**FIGURE 1-A3:**
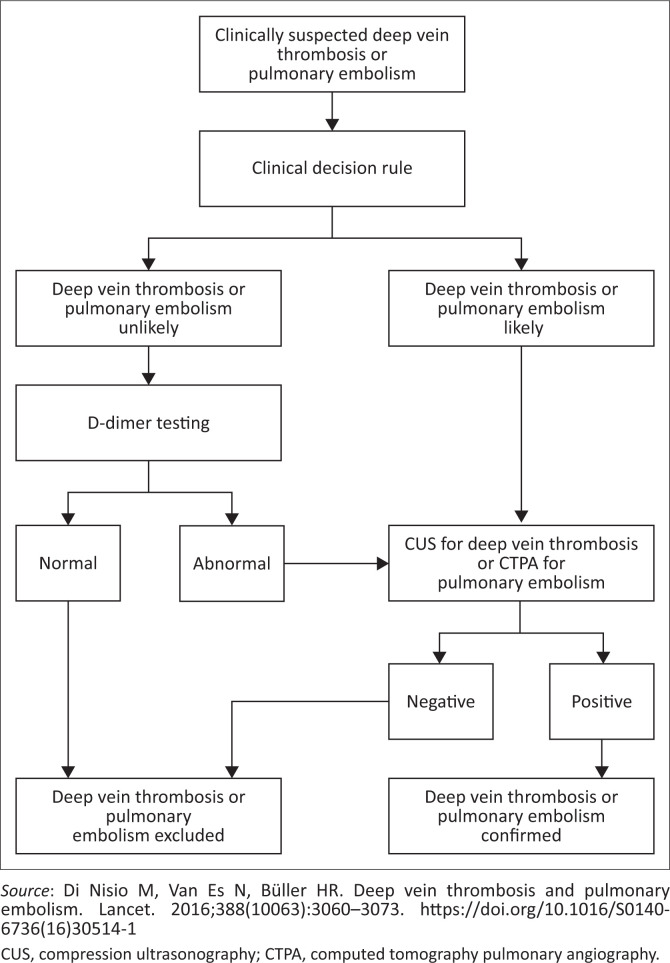
Diagnostic algorithm for suspected venous thromboembolism.

**FIGURE 1-A4:**
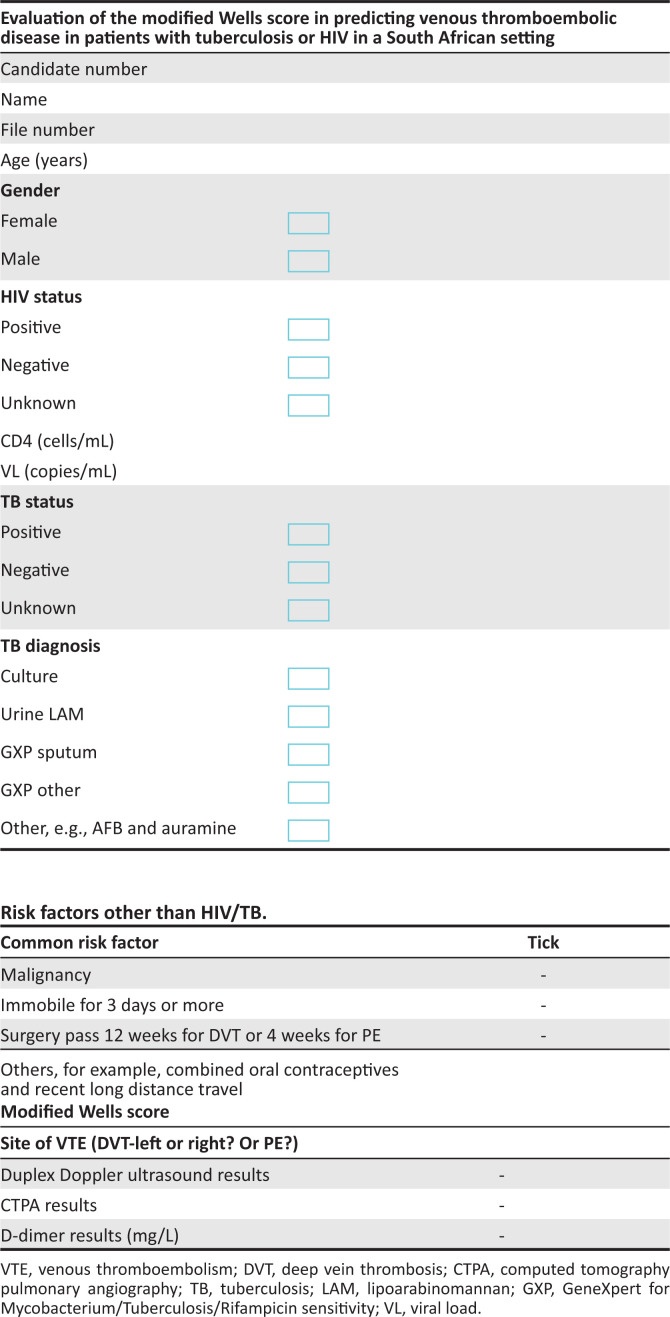
Data collection info sheet.

**FIGURE 1-A5:**
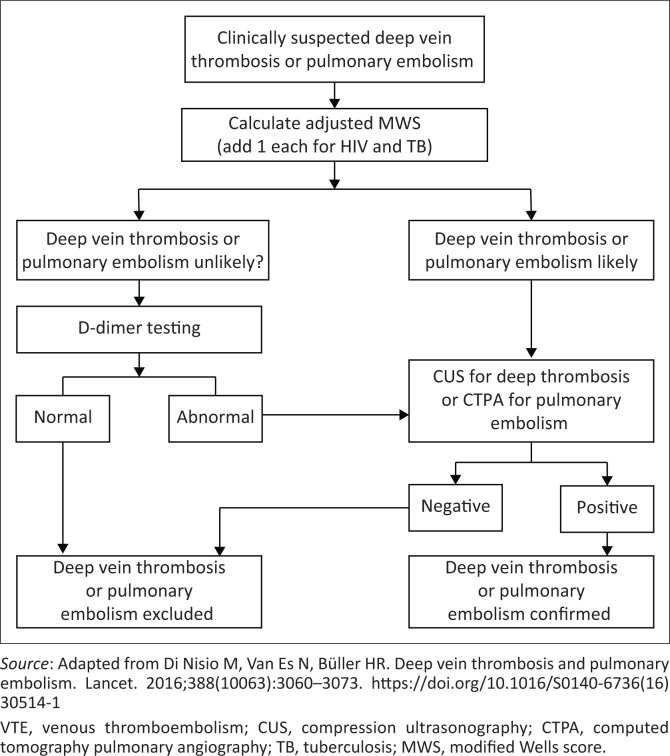
Diagnostic algorithm for suspected venous thromboembolism with adjusted modified Wells score for HIV/tuberculosis.
